# Association between *Loa loa* microfilaremia and anatomical hyposplenia in a rural area of the Republic of Congo: a population-based cross-sectional study

**DOI:** 10.1186/s40249-025-01277-w

**Published:** 2025-02-08

**Authors:** Charlotte Boullé, Elodie Lebredonchel, Jérémy T. Campillo, Valentin Dupasquier, Marlhand C. Hemilembolo, Sébastien D. S. Pion, Jean Claude Djontu, Ludovic Rancé, Philippe Souteyrand, François Missamou, Michel Boussinesq, Francine Ntoumi, Cédric B. Chesnais

**Affiliations:** 1 TransVIHMI, University of Montpellier, Inserm (National Institute of Health and Medical Research) Unité 1175, French National Research Institute for Sustainable Development (IRD), Montpellier, France; 2 Department of Infectious and Tropical Diseases, Montpellier University Hospital, 39 Av. Charles Flahault, 34090 Montpellier, France; 3 Department of Biochemistry, Paris Nord Val de Seine University Hospital-Bichat, Public Hospitals of Paris, Paris, France; 4 Department of Cardiology, Montpellier University Hospital, Montpellier, France; 5 National Onchocerciasis Control Program (PNLO), General Direction of Epidemiology and Disease Control in Brazzaville, Ministry of Health and Population, Brazzaville, Republic of Congo; 6 Congolese Foundation for Medical Research, Brazzaville, Republic of Congo; 7 Institute of Tropical Medicine, University of Tübingen, Tübingen, Germany; 8 Department of Anesthesiology and Critical Care Medicine, Montpellier University Hospital, Montpellier, France; 9 Department of Medical Imaging, European Hospital, Marseille, France; 10 Institut de Recherche pour le Développement (IRD), 911 Avenue Agropolis, 34394 Montpellier, France

**Keywords:** Loiasis, Spleen, Ultrasonographic examination, Malaria, Parasitic diseases, Splenomegaly, Hyposplenism

## Abstract

**Background:**

Data suggest excess mortality is associated with loiasis, which is endemic to Central Africa, although the underlying mechanisms remain unknown. We hypothesized that there could be an association between *Loa loa* microfilarial densities (MFD) and lower spleen volume (SV) due to micro-obstruction linked to circulating microfilariae (mf). This could result in functional hyposplenia and a higher burden of infections. Our objective was to investigate the impact of *L. loa* MFD and malaria on spleen’s bi-dimensional dimensions, volume, and parenchymal lesions.

**Methods:**

We included 981 participants aged 18–88 years in a cross-sectional study conducted in May–June 2022 in the Republic of the Congo. Centralized ultrasonographic examination was performed. The primary outcomes included SV, splenomegaly (cranio-caudal-distance ≥ 13 cm), and anatomical hyposplenia (AH) (SV ≤ 80, ≤ 110 or ≤ 150 cm^3^). Blood samples were analyzed for *L. loa* MFD, *Plasmodium-*PCR, Anti-*Plasmodium falciparum*-IgG, total IgM, sickle-cell disease status, and hematological abnormalities. Linear and logistic regressions were used to assess these associations.

**Results:**

Among 981 participants, 139 (14.1%) had splenomegaly, and 26 (2.7%) and 175 (17.8%) had SV ≤ 80 and ≤ 150 cm^3^, respectively. *L. loa* microfilariae were detected in 353 (35.6%) participants. A gradient effect was observed in each model, with the highest MFD (> 30,000 mf/ml) having the highest adjusted odds ratio of 17.94 (95% *CI*: 2.91–110.76, *P* = 0.002), 5.94 (95% *CI*: 1.40–25.17, *P* = 0.016), and 5.77 (95% *CI*: 1.95–17.12, *P* = 0.002) for SV ≤ 80, 110, and 150 cm^3^, respectively. Anti-*P. falciparum*-IgG levels were gradually associated with splenomegaly. Fourteen participants met the criterion for hyper-reactive malarial splenomegaly (HMS). Conversely, higher *L. loa* MFD was correlated with AH, with an attributable fraction of 25%, and the presence of splenic parenchymal lesions.

**Conclusions:**

This study provides a detailed description of spleen morphology and the factors influencing its size in a rural central African population. It demonstrates a strong association between *L. loa* MFD and reduced SV, suggesting that loiasis may lead to AH, and potentially to functional hyposplenia, with consequences such as increased susceptibility to bacterial infections. Malaria was associated with splenomegaly, with a figure of HMS consistent with estimates from other African countries.

**Graphical Abstract:**

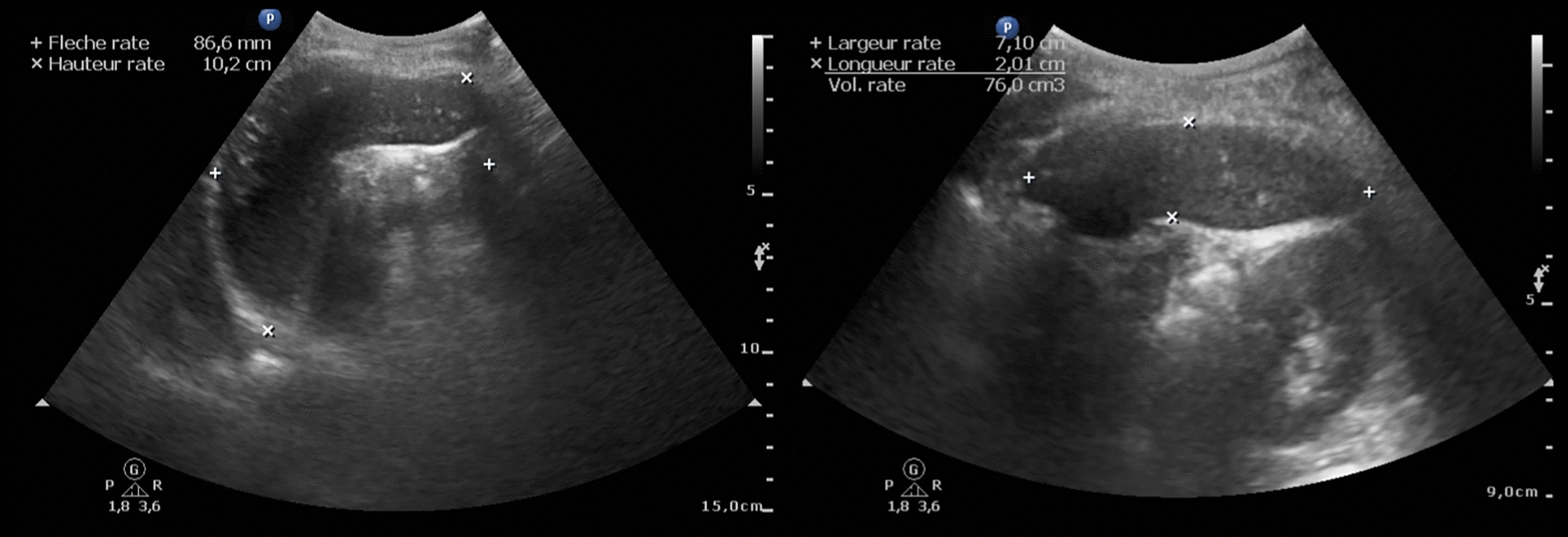

## Background

Loiasis, caused by the filarial worm *Loa loa*, is transmitted by tabanid flies (*Chrysops* sp.) in Central Africa and West Africa. Demographic data suggest that tens of millions of people are exposed and 15 million are actually infected [1]. A notable characteristic of loiasis is that female worms can produce millions of microfilariae (mf) circulating in the blood, with infected individuals potentially harboring very high microfilarial densities (MFD) exceeding 100,000 mf/ml of blood. Additionally, loiasis is a chronic infection as adult worms live up to 20 years, and the MFD of untreated individuals remains fairly stable over time [2–4]. Significant eosinophilia is commonly associated, which may contribute to coagulation and cardiac disorders [5, 6].

Although historically loiasis has been described as causing benign manifestations, such as pruritus, subconjunctival migration of the adult worms (“eye worm”), and transient episodes of angioedema (“Calabar swellings”), retrospective population-based cohort studies in Cameroon and the Republic of Congo demonstrated that individuals with high *L. loa* MFD had a significantly reduced life-expectancy and that the risk of premature death may be proportional to the individual’s MFD [7, 8]. Recent results show that loiasis has a significant burden of disease [9], and can induce complications affecting different organs such as the cardiovascular system, central nervous system, and kidneys [10–13], which may partially explain this excess mortality.

Although the possible presence of splenic nodules in patients with loiasis has been described for a long time [14], data on the impact of filariasis on the spleen are scarce [15]. It has been reported that the spleens of monkeys infected with *L. loa* present nodules that gradually disappear after an antifilarial treatment with diethylcarbamazine [16]. More recently, splenic lesions have been found in 8% of European cases and 1.9% of Gabonese cases of loiasis, respectively [17, 18]. The pathogenic processes underlying the development of these nodules remain unclear. However, several cases of hypoechoic splenic nodular lesions associated with granulomas containing mf have been reported in loiasis patients [14, 19]. In one case, treatment with ivermectin led to regression of splenic lesions [20]. In another patient, who died after receiving diethylcarbamazine treatment, foci of necrosis containing degenerated mf were found in the spleen [21].

In the context of polyparasitism among individuals living in Central Africa, particularly with the presence of malaria and hyperimmune tropical splenomegaly (also referred to as chronic malarial splenomegaly), which coexists with a significant prevalence of sickle cell disease, we aimed to consider loiasis as another potential factor that may have a deleterious effect on the spleen. Furthermore, sickle cell disease poses the dual challenge of splenomegaly during crises and secondary involution due to multiple infarctions; the ultimate consequence being the decreased functionality of the spleen against infections.

A meticulous examination of spleen volume (SV) was undertaken to test the hypothesis that *L. loa* MFD might be associated with smaller SV, owing to alteration of the reticuloendothelial system. Our objective was to characterize the association between *L. loa* microfilaremia and lower SV. We also aimed to describe the splenic ultrasound characteristics in a general population residing in rural areas of the Republic of Congo, and to characterize factors associated with anomalies in volume, size [understood here as a bi-dimensional measurement, namely height, length, width, and cranio-caudal distance (CCD)], and the presence of lesions, including loiasis and malaria.

## Methods

### Study area and population

This cross-sectional study nested within a cohort, conducted between May and June 2022, included 991 participants aged between 18–88 years, residing in 21 villages proximate to Sibiti, Republic of Congo. Participants were evaluated for *L. loa* microfilaremia in 2021 [22]. Individuals diagnosed with blood *L. loa* mf in 2021 were matched for sex and age (± 5 years) with two amicrofilaremic individuals from the same village. The sample size for the underlying cohort study had been calculated (990) to show an increase in the risk of malaria and pneumonia of × 1.4 and × 2.4 for microfilaremic vs amicrofilaremic individuals, with a power of 80%. In the present cross-sectional study, the sample size was not predetermined to detect differences in spleen volume due to a lack of prior data for calculation. However, with a sample size of 990, and assuming an 8% prevalence of ultrasonographic lesions in microfilaremics [17] and 3% in amicrofilaremics, the study had a calculated power of 89.6% to detect such a difference. Participation in the study was contingent upon informed consent. The study was approved by the Ethics Committee of the Congolese Foundation for Medical Research (036/CIE/FCRM/2022) and by the national administrative authorities of Congo (376/MSP/CAB/UCPP-21).

### Laboratory procedures

#### Screening of parasitic diseases

Each patient had 50 µl of blood collected by finger-prick using a lancet between 10:00 am and 4:00 pm to prepare a thick blood smear (TBS), stained with Giemsa and examined under a microscope at 100 × magnification by experienced technicians to count the *L. loa* and *Mansonella perstans* mf. Each TBS was read twice and the arithmetic mean of the counts was used.

Venous blood was collected in a heparinized tube to perform a *L. loa* pan-IgG Rapid Diagnostic Test (pan-IgG-RDT, Drugs & Diagnostics for Tropical Diseases, San Diego, California), and the test line was compared to a 10-color scale [23].

The tests, including anti-*P. falciparum* IgG (µg/ml) by ELISA [24] and identification of *Plasmodium* by nested PCR [25], were performed at the Congolese Foundation for Medical Research laboratory (Brazzaville, Republic of Congo). Total serum IgM were measured by AgoraCare (Brazzaville, Republic of Congo).

Participant’s past exposure to *Onchocerca volvulus* was assessed using an Ov16 Rapid Diagnostic Test (Biplex *L. loa*/Ov16 RDT; Drugs & Diagnostics for Tropical Diseases, San Diego, California). Two skin snips were collected from each patient with positive Ov16 RDT using a 2 mm Holth-type corneoscleral punch and incubated in saline at room temperature for 24 h. Emerged mf were counted under a microscope, and the individuals’ *O. volvulus* MFD, expressed as mf per snip, were calculated using the arithmetic mean of the counts from the two snips.

Analysis for *Schistosoma haematobium* infection was conducted exclusively on individuals exhibiting hematuria upon dipstick assessment, employing urine filtration, Lugol staining, and subsequent microscopic examination.

Infections with soil-transmitted helminths (STHs) were identified by microscopic examination of morning stool specimens shipped to the laboratory within 6 h, and processed immediately or after overnight storage at 6 ℃. Using the Kato-Katz method, a thick smear was prepared from each stool sample and these smears were examined under a microscope at 40 × magnification.

#### Hematological assessment

Sickle cell disease (SSD) status (HbAA, HbAS, and HbSS) was assessed using a rapid test (HemoTypeSCTM, HT401RUO-USA) on blood collected in EDTA tubes [26–28].

Differential white blood cell counts were performed with HemoCue WBC DIFF (HemoCue WBC DIFF system, HemoCue AB, Ängelholm, Sweden) device.

Blood smears for hematological abnormalities and count of Howell Jolly Bodies (HJB) were performed using RAL 555 kits (RAL Diagnostics, CELLAVISION, Martillac, France) and read by a medical biologist.

### Collection of socio-demographic and covariables data

For each participant, data on age, sex, occupation, anthropometric measurements, tobacco use, and history of medical conditions were collected. Additionally, we gathered information on the number of eye worm (Ew) episodes during their lifetime. In absence of validated method to evaluate the number of adult worms present in a given individual, we assumed that the number of such episodes, which can be readily assessed, might be a proxy of adult worm burden.

### Outcome: spleen evaluation using ultrasonography (US)

The US examination of the spleen was performed by experienced physicians using a CX-50 device (Philips Medical Systems, Suresnes, France). Measurements included height, width, length, CCD, and volume. A second reading of the digital images (DICOM files) was performed by a radiologist to confirm and characterize spleen parenchymal lesions (single or multiple, hyperechoic or hypoechoic, nodular or ill-defined, homogeneous or heterogeneous, calcific or cystic nature). Nodules were classified as micronodules when their size was < 10 mm, and as macronodules when they were ≥ 10 mm. Splenomegaly was defined as CCD ≥ 13 cm. Assessing spleen size and volume is challenging due to the lack of a universally accepted gold standard in field settings. CCD was used due to its good correlation with CT findings [29], with a threshold of 13 cm to improve specificity. We introduce here the term of anatomical hyposplenia (AH)—in mirror to that of splenomegaly. In the absence of norms in our study population, and more generally in the absence of validated cut-offs to define a low spleen volume, we chose to use three cutoffs according to the SV: ≤ 80 cm^3^ associated with excess mortality in infections by *Streptococcus pneumoniae* [30], ≤ 110 cm^3^ which was -1 standard deviation (*SD*) of the SV in our population, and ≤ 150 cm^3^ which is considered the lower bound of normal spleen size [29]. Last, hyper-reactive malarial splenomegaly (HMS) was defined as ‘possible’ in the association of CCD ≥ 13 cm with total serum IgM > 2.5 g/L or as ‘probable’ in the association of CCD ≥ 13 cm with total serum IgM > 3.5 g/L, and anti-plasmodial IgG > 85 µg/ml (upper quartile) [31, 32].

### Statistical analysis

Linear and logistic regressions were used to assess the associations between SV, splenomegaly, small spleen size (≤ 80, 110 or 150 cm^3^), and splenic parenchymal lesions with explanatory variables. Final models were obtained through stepwise manual descending procedure using Wald test with a significant level of *P* < 0.15. Several explanatory variables were included: age (continuous), sex (male vs. female), body mass index (BMI; continuous), history of cerebral malaria, number of Ew episodes during lifetime (0, 1–5, 6–10, > 10), neutropenia (< 1500/µl), lymphopenia (< 1200/µl [33]), HJB (presence or absence), loiasis RDT (intensity categorized in 0, 1–5, > 5), SSD status (HbAA, HbAS, HbSS), *Ascaris lumbricoides*, *Trichuris trichiura* (presence or absence), *L. loa* MFD (0, 1–1999 mf/ml, 2000–7999 mf/ml, 8000–29,999 mf/ml and ≥ 30,000 mf/ml, that are standard cut-offs in the literature [34]), anti-*Plasmodium*-IgG (quartiles), PCR *Plasmodium* (positive or negative). Tobacco use was included a priori as it is common in health assessments and a risk factor mainly for onco-hematological conditions that can be in turn associated with changes in spleen morphology. STH are associated with splenomegaly, mostly in children and were also included in the analysis to eliminate potential confounders. A history of cerebral malaria was included as we aimed at looking if there was a relation with hyperimmune splenomegaly, and as malaria is a frequent morbidity in the area.

Finally, the population attributable fraction (PAF)—representing the proportion of the AH burden that could be eliminated if *L. loa* microfilaremia was eliminated—were extracted from final models with *L. loa* status as a binary variable (microfilaremic vs. amicrofilaremic).

All statistical analyses were performed using Stata 17 (StatCorps LP, College Station, Texas, USA).

## Results

### Description of the study population

Among the 991 participants enrolled in the parent study, 981 (612 men, 369 women) underwent ultrasound examination. Of these, the spleen could not be visualized in 10 patients, and for them, a zero value was included for all spleen-related measures in the analysis. The socio-demographical and clinical characteristics of the 981 participants are described in Table 1. Splenomegaly (CCD ≥ 13 cm) was observed in 139 out of 981 (14.3%) participants. A total of 26 out of 977 (2.7%), 61 out of 977 (6.2%), and 175 out of 977 (17.9%) individuals had SV ≤ 80, ≤ 110 and ≤ 150 cm^3^, respectively. Parenchymal lesions on US examination were noted for 42 out of 981 (4.3%) participants, with four of them exhibiting two types of lesions (total number of lesions *n* = 46). A detailed description of the lesions is given in Table 2. *L. loa* microfilaremia was found in 353 out of 981 (35.6%) participants, with a median MFD of 2440 mf/ml. One patient had chronic lymphocytic leukemia (SV: 848 cm^3^; CCD: 11.9 cm), five had circulating lymphoplasmocytes (SV: 258 and 37 cm^3^; CCD: 11.2 and 5.6) or plasmocytes (SV: 194, 263 and 273 cm^3^; CCD: 12.1, 9.3 and 9.5 cm). Among participants, there were no cases of hookworm infection or infection with *S. mansoni*. No schistosomiasis was found among the 94 individuals with hematuria (11 traces/+ and 83 ++ / +++). Only 6 out of 981 individuals (0.6%) had *M. perstans* mf in their blood (range: 20–660 mf/ml). Twenty-two individuals (2.2%) tested positive for the Ov16 RDT, and none of them had *O. volvulus* mf in the skin snips.

**Table 1:** Characteristics of the study population

	*n* = 981
Sex	
Female	369 (37.5%)
Male	612 (62.5%)
Age (years)	52 (40–62)
Height (cm) (*n* = 980)	164 (157–169)
Weight (kg) (*n* = 980)	55 (50–61)
BMI (kg m^−2^) (*n* = 980)	20.6 (19.0–22.4)
Main occupation	
Farmer	773 (78.8%)
No activity	55 (5.6%)
Healthcare worker	12 (1.2%)
Other	141 (14.4%)
Smoking (*n* = 973)	179 (18.4%)
Hepatomegaly on physical examination (*n* = 980)	57 (5.8%)
Splenomegaly on physical examination (*n* = 980)	115 (11.7%)
Complete blood count (per µl) (*n* = 909)	
Neutrophils, median (*IQR*)	2400 (1900–3200)
< 1500 per µl	109 (12.0%)
Lymphocytes, median (*IQR*)	2700 (2100–3300)
< 1200 per µl	20 (2.2%)
Eosinophils	900 (400–1700)
Monocytes	300 (200–400)
Hematological blood smear abnormality (*n* = 979)	
Codocytes	14 (1.4%)
Chronic lymphocytic leukemia	1 (0.1%)
Lymphoplasmocytes	2 (0.2%)
Plasmocytes	3 (0.3%)
Presence of Howell-Jolly Bodies (*n* = 972)	171 (17.6%)
Median % of red cells with HJB	1.7% (1.7–3.3%)
< 2%	101 (59.1%)
> 2%	70 (40.9%)
Sickle cell diseases status (*n* = 971)	
AA	754 (77.9%)
AS	217 (22.1%)
Medical conditions	
History of cerebral malaria	111 (11.3%)
Soil transmitted helminthiases	
Hookworm infection (*n* = 766)	0 (0%)
Trichuriasis (*n* = 767)	207 (26.8%)
Ascaridiasis (*n* = 765)	329 (43.0%)
Mild (< 5000 eggs per g of stool)	310 (94.1%)
Moderate (5000–49,999 eggs per g of stool)	19 (5.8%)
Severe (> 50,000 eggs per g of stool)	0
Spleen metrics	
Cranio-caudal distance (cm) (*n* = 971)	9.8 (8.0–11.9)
< 13 cm (including 10 atrophic^a^)	842 (85.7%)
≥ 13 cm	139 (14.3%)
Height (cm) (*n* = 971)	10.5 (9.4–11.9)
Length (cm) (*n* = 968)	4.8 (4.2–5.5)
Width (cm) (*n* = 967)	9.2 (8.1–10.3)
Volume (*n* = 967)	239 (177–331)
≤ 80 cm^3^ (including 10 atrophic^a^)	26/977 (2.7%)
≤ 110 cm^3^ (including 10 atrophic^a^)	61/977 (6.2%)
≤ 150 cm^3^ (including 10 atrophic^a^)	175/977 (17.9%)
150–300 cm^3^	493/977 (50.5%)
> 300 cm^3^	309/977 (31.6%)
*L. loa* microfilaremia	
Presence	347 (35.4%)
*L. loa* Microfilarial density (mf per ml), median (*IQR*)	0 (0–580)
0	634 (64.6%)
1–1999	164 (16.7%)
2000–7999	86 (8.8%)
8000–29,999	79 9.0%)
> 30,000	18 (1.8%)
Microfilarial density in positive individuals, median (*IQR*)	2440 (400–8840)
Number of episodes of eyeworm during life (*n* = 811)	
0	424 (43.2%)
1–5	170 (17.3%)
6–10	140 (14.3%)
> 10	77 (7.9%)
Pan-IgG-RDT loiasis (intensity from 0 to 10) (*n* = 963)	
0	57 (5.8%)
1–4	504 (51.4%)
5–6	358 (36.5%)
> 6	44 (4.5%)
Blood smear positive for *Plasmodium* (*n* = 979)	13 (1.3%)
*Plasmodium* PCR (*n* = 908)	
Negative	472 (52.0%)
Positive	436 (48.0%)
* P. falciparum*	378 (41.6%)
* P. ovale*	42 (4.6%)
* P. malariae*	104 (11.5%)
Anti-*Plasmodium* IgG (µg per ml), median (IQR), (*n* = 904)	68 (53–86)
Total serum IgM (g/l) among 126 of 139 individuals with CCD ≥ 13 cm^a^	2.2 (1.3–3.8)
> 2.5 g/L	52 (41.3%)
> 3.5 g/L	33 (26.2%)

Data are presented as number (percentage) or median (*IQR*)

*BMI* body mass index, *IQR* interquartile range, *HJB* Howell-Jolly Bodies, *RDT* rapid diagnostic test, *PCR* polymerase chain reaction, *CCD* cranio-caudal distance

^a^Atrophic spleen is defined as impossibility to identify the spleen through careful ultrasonographic examination

**Table 2:** Description of the 46 spleen parenchymal lesions observed in *n* = 42 patients with spleen lesions on ultrasonographic examination

Type of lesions	*n* (%)
Calcifications	7 (15.2%)
Single	3 (6.5%)
Multiple	3 (6.5%)
Probable Gamna-Gandy bodies	1 (2.2%)
Nodular lesions	24 (52.2%)
Micronodular	2 (4.3%)
Multiple hyperechoic	2 (4.3%)
Macronodular	22 (47.8%)
Single hypoechoic homogeneous	5 (10.9%)
Multiple hypoechoic homogeneous	3 (6.5%)
Single hyperechoic homogeneous	5 (10.9%)
Multiple hyperechoic homogeneous	3 (6.5%)
Single hyperechoic heterogeneous	6 (13%)
Tissular	8 (17.4%)
Single ill-defined hypoechoic lesion	1 (2.2%)
Single ill-defined hyperechoic heterogeneous lesion	4 (8.7%)
Multiple ill-defined hyperechoic heterogeneous lesions	2 (4.3%)
Diffuse heterogeneous lesions of the whole parenchyma	1 (2.2%)
Liquid (kystic)	7 (15.2%)
Single	6 (13%)
Multiple	1 (2.2%)

*n* = 4 patients had two different types of lesions on ultrasonographic examination

### Factors influencing spleen volume and splenomegaly (Table 3)

Along with age-related atrophy (ß = − 2.17; 95% *CI*: − 2.86, − 1.48, *P* < 0.001), SV was larger in males than in females (+ 35.11 cm^3^; 95% *CI*: 14.4, 55.82, *P* = 0.001) and in people with higher BMI (+ 4.59 cm^3^ per kg·m^−2^; 95% *CI*: 1.51, 7.68, *P* = 0.004); and HJB presence was associated with lower SV (− 26.48 cm^3^; 95% *CI*: − 52.03, − 0.93, *P* = 0.042) (Table 3). Malarial seropositivity titer in IgG was associated with enlarged SV with a gradient effect (global Wald test *P* < 0.001): + 20.43 (95% *CI*: − 7.90, 48.76), + 43.63 (95% *CI*: 13.12, 74.15), + 66.70 (95% *CI*: 37.07, 96.33) for 50–70, 70–85, and > 85 µg per ml, respectively. Although lacking a significant effect, the *Plasmodium* PCR had the same tendency. Conversely, the *L. loa* MFD was associated with lower SV with a gradient effect [Wald global test *P* = 0.004; − 40.94 cm^3^ for those with *L. loa* MFD > 30,000 mf/ml (*P* = 0.277)], compared to amicrofilaremic ones). Individuals with splenic parenchymal lesions had a mean enlarged SV of 58.23 cm^3^ (95% *CI*: 10.14, 106.31, *P* = 0.018).

**Table 3:** Factors associated with spleen volume in cm^3^ or splenomegaly defined as cranio-caudal distance ≥ 13 cm in univariate and multivariable analysis

Variable	Spleen volume (cm^3^)				Splenomegaly^#^			
	Saturated model		Final model		Saturated model		Final model	
	ß-coefficient [95% *CI*]	*P*	ß-coefficient [95% *CI*]	*P*	*aOR* [95% *CI*]	*P*	*aOR* [95% *CI*]	*P*
Age	− 2.16 [− 2.88, − 1.43]	< 0.001	− 2.17 [− 2.86, − 1.48]	< 0.001	0.98 [0.96, 0.99]	0.003	0.98 [0.97, 0.99]	0.004
Sex (Ref.: female)	34.05 [12.70, 55.40]	0.002	35.11 [14.40, 55.82]	0.001	1.14 [0.76, 1.73]	0.527		
BMI	4.80 [1.66, 7.93]	0.003	4.59 [1.51, 7.68]	0.004	1.04 [0.98, 1.10]	0.198		
History of neurological malaria (Ref.: No)	− 5.20 [− 36.70, 26.31]	0.746			1.14 [0.63, 2.03]	0.671		
HbAS SCD (Ref.: HbAA)	− 5.17 [− 29.40, 19.06]	0.676			0.66 [0.40, 1.10]	0.112	0.66 [0.40, 1.09]	0.101
Neutropenia	29.90 [− 2.67, 62.47]	0.072	29.23 [− 1.99, 60.45]	0.066	1.46 [0.83, 2.57]	0.187		
Lymphopenia	32.73 [− 38.07, 103.53]	0.364			1.38 [0.64, 2.99]	0.216	1.57 [0.91, 2.68]	0.103
*Ascaris lumbricoides* (Ref.: absence)	13.92 [− 10.25, 38.08]	0.259	17.4 [− 4.98, 39.79]	0.127	2.04 [0.66, 6.32]	0.458		
*Trichuris trichiura* (Ref.: absence)	10.66 [− 16.63, 37.96]	0.443			1.20 [0.75, 1.92]	0.518		
Number of Eye worm episodes (Ref.: 0)								
1–5	6.30 [− 22.03, 34.63]	0.663			1.19 [0.71, 2.00]	0.739		
6–10	− 3.02 [− 33.90, 27.86]	0.848			0.91 [0.52, 1.58]	0.702		
> 10	1.47 [− 37.63, 40.56]	0.941			1.12 [0.63, 2.01]	0.884		
MD	− 31.76 [− 84.89, 21.37]	0.241			0.95 [0.44, 2.02]	0.069		
Pan-IgG-RDT loiasis (Ref.: 0)								
1–5	8.11 [− 35.23, 51.45]	0.714			0.42 [0.16, 1.07]	0.885		
> 5	− 0.18 [− 48.21, 47.84]	0.994			0.94 [0.41, 2.15]	0.709		
HBJ (Ref. absence)	− 26.26 [− 52.39, − 0.13]	0.049	− 26.48 [− 52.03, − 0.93]	0.042	0.84 [0.33, 2.11]	0.003	0.4 [0.21, 0.74]	0.004
Anti-*Plasmodium* IgG titer (Ref.: < 50 µg per ml)				< 0.001^*^				0.003^*^
50–70	21.61 [− 7.59, 50.82]	0.147	20.43 [− 7.90, 48.76]	0.157	0.38 [0.20, 0.73]	0.211	1.49 [0.79, 2.81]	0.217
70–85	40.38 [8.91, 71.84]	0.012	43.63 [13.12, 74.15]	0.005	1.51 [0.79, 2.89]	0.009	2.42 [1.28, 4.58]	0.007
> 85	63.77 [33.28, 94.25]	< 0.001	66.70 [37.07, 96.33]	< 0.001	2.37 [1.24, 4.56]	< 0.001	3.03 [1.62, 5.66]	0.001
*Plasmodium* PCR (Ref.: absence)	17.73 [− 3.27, 38.73]	0.098	16.31 [− 4.20, 36.81]	0.119	3.15 [1.67, 5.97]	0.621		
Splenic US parenchymal lesion (Ref.: absence)	57.06 [7.48, 106.63]	0.024	58.23 [10.14, 106.31]	0.018	0.90 [0.60, 1.35]	0.012	2.58 [1.18, 5.61]	0.017
*L. loa* Microfilarial density (Ref.: 0 mf per ml)				0.004^*^				0.021^*^
1–2000	− 23.37 [− 50.73, 3.99]	0.09	− 24.35 [− 51.13, 2.43]	0.075	2.78 [1.25, 6.19]	0.029	0.53 [0.30, 0.93]	0.028
2001–8000	− 51.83 [− 88.08, − 15.58]	0.005	− 51.80 [− 87.61, − 15.99]	0.005	0.52 [0.29, 0.94]	0.059	0.48 [0.22, 1.06]	0.070
8001–30,000	− 55.09 [− 93.43, − 16.75]	0.005	− 52.28 [− 89.11, − 15.45]	0.005	0.46 [0.20, 1.03]	0.095	0.47 [0.20, 1.07]	0.073
> 30,000	− 38.19 [− 112.75, 36.37]	0.32	− 40.94 [− 114.88, 33.00]	0.277	0.48 [0.21, 1.13]	0.543	0.59 [0.13, 2.70]	0.500

A category of missing data (MD) was included for variables neutropenia, thrombopenia, *Ascaris*, *Trichuris*, IgG, PCR, but was not significant and is not presented for the sake of clarity. *Ref* reference category, *BMI* body mass index, *SCD* sickle cell disease, *RDT* rapid diagnostic test, *HJB* Howell-Jolly Bodies, *PCR* polymerase chain reaction, *US* ultrasound, *CI* confidence interval, *aOR* adjusted odds ratio

^#^Splenomegaly was defined as cranio-caudal distance > 13 cm ^*^Wald global test *P*-value

In the final model, splenomegaly (CCD ≥ 13 cm) was inversely associated with age (*aOR* = 0.98; 95% *CI*: 0.97, 0.99, *P* = 0.004), and associated with the absence of HJB (*aOR* = 0.4; 95% *CI*: 0.21, 0.74, *P* = 0.004). There appeared to be a non-statistically significant association of splenomegaly with the absence of SCD trait (*aOR* = 0.66; 95% *CI*: 0.40, 1.09, *P* = 0.101), and the presence of a lymphopenia (*aOR* = 1.57; 95% *CI*: 0.91, 2.68, *P* = 0.103). Malarial seropositivity was also gradually associated with CCD ≥ 13 cm, with *aORs* ranging from 1.49 to 3.03 (*P* = 0.003) for those with the highest serological titers. The reverse was observed with the *L. loa* MFD (global Wald test *P* = 0.021): 0.53 (95% *CI*: 0.30, 0.93), 0.48 (95% *CI*: 0.22, 1.06), 0.47 (95% *CI*: 0.20, 1.07), and 0.59 (95% *CI*: 0.13, 2.70) for 1–2000, 2000–8000, 8000–30,000, and > 30,000 mf/ml, respectively.

Notably, among the patients with CCD ≥ 13 cm, 2 had codocytes, 1 had activated lymphocytes, and 4 had *P. falciparum* trophozoites.

Among the 139 participants who had spleen CCD ≥ 13 cm, median total serum IgM level (determined for 126 individuals) was 2.2 g/L (interquartile range, *IQR*: 1.3–3.8). Total IgM > 2.5 g/L combined with CCD ≥ 13 cm (‘possible’ HMS) was seen in 52 participants (5.3%), including 35 males (60.3%); their median age was 56 years (*IQR*: 45–65). Total IgM levels exceeding the higher threshold (> 3.5 g/L), anti-*Plasmodium* IgG levels > 85 µg/ml, and CCD ≥ 13 cm (‘probable’ HMS) was combined in 14 participants (1.4%), including 9 males (64.3%); their median age was 57 years (*IQR*: 45–69). The latter group of 14 participants, exhibited significantly higher lymphocytes levels (Wilcoxon-Mann–Whitney rank test *P* = 0.04) as compared to controls (median 3300 per µl, *IQR*: 2500–4500 versus 2700, *IQR*: 2100–3300). None had hematological malignancy or plasmodial infection on the blood smear. *Plasmodium* PCR was positive for 19/52 (36.5%) subjects with ‘possible’ HMS and 3/14 (21.4%) subjects with ‘probable’ HMS.

### Factors associated with anatomical hyposplenia (Table 4)

Age was positively associated with AH [*aORs* = 1.05; (95% *CI*: 1.01, 1.09, *P* = 0.007), 1.04 (95% *CI*: 1.02, 1.06, *P* = 0.001), 1.04 (95% *CI*: 1.03, 1.06, *P* < 0.001), for < 80, 110, and 150 cm^3^, respectively], whereas BMI was negatively associated with AH [*aORs* = 0.79; (95% *CI*: 0.67, 0.94, *P* = 0.007), 0.86 (95% *CI*: 0.77, 0.95, *P* = 0.003), 0.91 (95% *CI*: 0.86, 0.97, *P* = 0.002), for < 80, 110, and 150 cm^3^, respectively]. Male sex was negatively associated with AH only in the < 150 cm^3^ model (*aOR* = 0.55; 95% *CI*: 0.38, 0.80, *P* = 0.001). HJB presence was solely associated with SV ≤ 80 cm^3^ with *aOR* of 3.85 (95% *CI*: 1.55, 9.56, *P* = 0.004). When considering the HBJ as a categorical variable [0 (reference), ≤ 2%, and > 2%], the final model (for SV ≤ 80 cm^3^) retained the same variables, with a gradient effect for HBJ (< 2%: *aOR* = 3.43; 95% *CI*: 1.21, 9.68, *P* = 0.020; > 2%: *aOR* = 4.25, 95% *CI*: 1.29, 16.63,* P* = 0.038). A PCR positive *Plasmodium* infection [*aORs* = 0.44; 95% *CI*: 0.18, 1.12, *P* = 0.086), 0.62 (95% *CI*: 0.35–1.12, *P* = 0.116), 0.52 (95% *CI*: 0.35–0.76, *P* = 0.001), for < 80, 110, and 150 cm^3^, respectively], and higher anti-*P. falciparum* serological levels (> 85 µg per ml) were inversely associated with lower SV [*aORs* = 0.19; 95% *CI*: 0.04, 0.94, *P* = 0.042), 0.52 (95% *CI*: 0.30, 0.91, *P* = 0.022), for < 80, and 150 cm^3^, respectively]. Higher *L. loa* MFD were associated with increased risk of low SV. A gradient effect was observed in each model, with the highest MFD (> 30,000 mf/ml) having the highest *aOR* of 17.94 (95% *CI*: 2.91, 110.76, *P* = 0.002), 5.94 (95% *CI*: 1.40, 25.17, *P* = 0.016), and 5.77 (95% *CI*: 1.95, 17.12, *P* = 0.002) for SV ≤ 80, 110, and 150 cm^3^, respectively.

**Table 4:** Factors associated with anatomical hyposplenia defined as spleen volume < 80, 110, or 150 cm^3^ in univariate and multivariable analysis

Variable	< 80 cm^3^				< 110 cm^3^				< 150 cm^3^			
	Saturated model		Final model		Saturated model		Final model		Saturated model		Final model	
	*aOR* [95% *CI*]	*P*	*aOR* [95% *CI*]	*P*	*aOR* [95% CI]	*P*	*aOR* [95% *CI*]	*P*	*aOR* [95% *CI*]	*P*	*aOR* [95% *CI*]	*P*
Age	1.05 [1.01, 1.09]	0.011	1.05 [1.01, 1.09]	0.007	1.04 [1.02, 1.07]	< 0.001	1.04 [1.02, 1.06]	0.001	1.04 [1.03, 1.06]	< 0.001	1.04 [1.03, 1.06]	< 0.001
Sex (Ref.: female)	0.89 [0.36, 2.23]	0.810			0.58 [0.32, 1.05]	0.070	0.64 [0.36, 1.11]	0.110	0.54 [0.37, 0.78]	0.001	0.55 [0.38, 0.80]	0.001
BMI	0.76 [0.63, 0.92]	0.004	0.79 [0.67, 0.94]	0.007	0.85 [0.76, 0.95]	0.003	0.86 [0.77, 0.95]	0.003	0.91 [0.85, 0.96]	0.002	0.91 [0.86, 0.97]	0.002
History of neurological malaria (Ref.: No)	0.70 [0.14, 3.53]	0.667			1.36 [0.58, 3.18]	0.481			1.00 [0.56, 1.78]	0.999		
HbAS SCD (Ref.: HbAA)	1.10 [0.38, 3.17]	0.865			0.61 [0.29, 1.31]	0.204			0.68 [0.43, 1.09]	0.107	0.69 [0.44, 1.09]	0.109
Neutropenia	1.09 [0.27, 4.42]	0.903			1.10 [0.46, 2.62]	0.834			0.70 [0.38, 1.31]	0.267		
Lymphopenia	–				-				0.16 [0.02, 1.36]	0.094	0.13 [0.02, 1.07]	0.13 [0.02, 1.07]
*Ascaris lumbricoides*	0.84 [0.28, 2.53]	0.755			0.99 [0.51, 1.91]	0.971			0.86 [0.56, 1.32]	0.496		
*Trichuris trichiura*	1.23 [0.39, 3.84]	0.728			1.34 [0.66, 2.71]	0.415			1.11 [0.69, 1.79]	0.660		
Number of Eye worm episodes (Ref.: 0)		0.258^*^				0.345^*^				0.482^*^		
1–5	0.47 [0.11, 2.03]	0.312			0.73 [0.31, 1.74]	0.476			0.86 [0.50, 1.45]	0.561		
6–10	1.68 [0.49, 5.77]	0.411			1.19 [0.53, 2.68]	0.681			1.31 [0.78, 2.19]	0.308		
> 10	1.78 [0.46, 6.97]	0.406			1.88 [0.78, 4.56]	0.162			1.44 [0.76, 2.7]	0.264		
MD	0.28 [0.04, 1.81]	0.181			0.51 [0.12, 2.15]	0.360			0.82 [0.32, 2.12]	0.682		
Pan-IgG-RDT loiasis (Ref.: 0)		0.010^*^				0.001^*^				0.007^*^		
1–5	0.25 [0.08, 0.8]	0.020	0.24 [0.08, 0.76]	0.014	0.33 [0.14, 0.75]	0.008	0.32 [0.15, 0.71]	0.005	0.38 [0.20, 0.73]	0.003	0.39 [0.21, 0.74]	0.004
> 5	0.03 [0, 0.37]	0.006	0.04 [0, 0.43]	0.008	0.08 [0.02, 0.34]	0.001	0.11 [0.03, 0.38]	0.001	0.31 [0.14, 0.67]	0.003	0.34 [0.16, 0.72]	0.005
HBJ (Ref.: absence)	3.66 [1.43, 9.39]	0.007	3.85 [1.55, 9.56]	0.004	1.30 [0.64, 2.66]	0.469			1.28 [0.81, 2.03]	0.296		
Anti-*Plasmodium* IgG titer (Ref.: < 50 µg per ml)		0.149^*^		0.144^*^		0.187^*^				0.116^*^		0.119^*^
50–70	1.11 [0.31, 3.97]	0.874	1.15 [0.36, 3.65]	0.816	0.84 [0.37, 1.91]	0.678			0.87 [0.51, 1.46]	0.589	0.85 [0.51, 1.42]	0.528
70–85	0.83 [0.20, 3.50]	0.802	0.86 [0.23, 3.28]	0.827	0.60 [0.24, 1.51]	0.275			0.88 [0.50, 1.56]	0.670	0.87 [0.50, 1.52]	0.629
> 85	0.16 [0.03, 0.90]	0.037	0.19 [0.04, 0.94]	0.042	0.41 [0.16, 1.01]	0.051			0.53 [0.30, 0.92]	0.023	0.52 [0.30, 0.91]	0.022
*Plasmodium* PCR (Ref.: absence)	0.44 [0.17, 1.15]	0.096	0.44 [0.18, 1.12]	0.086	0.57 [0.31, 1.05]	0.069	0.62 [0.35, 1.12]	0.116	0.52 [0.35, 0.77]	0.001	0.52 [0.35, 0.76]	0.001
Splenic US parenchymal lesion (Ref.: absence)	0.56 [0.06, 5.18]	0.606			0.48 [0.10, 2.40]	0.373			0.43 [0.15, 1.20]	0.107	0.42 [0.15, 1.18]	0.099
*L. loa* Microfilarial density (Ref.: 0 mf per ml)		0.031^*^		0.037^*^		0.190^*^		0.085^*^		0.012^*^		0.002^*^
1–2000	1.74 [0.56, 5.43]	0.343	1.91 [0.63, 5.82]	0.257	2.11 [1.03, 4.32]	0.041	1.91 [0.95, 3.82]	0.068	1.37 [0.84, 2.23]	0.207	1.32 [0.82, 2.15]	0.258
2001–8000	1.92 [0.46, 8.09]	0.372	1.99 [0.50, 7.96]	0.332	1.62 [0.61, 4.32]	0.335	1.62 [0.63, 4.14]	0.315	2.06 [1.12, 3.79]	0.020	2.17 [1.19, 3.94]	0.011
8001–30,000	1.24 [0.22, 6.94]	0.804	1.34 [0.27, 6.65]	0.718	1.25 [0.42, 3.69]	0.691	1.34 [0.49, 3.72]	0.569	1.70 [0.88, 3.30]	0.114	1.92 [1.02, 3.63]	0.044
> 30,000	21.96 [3.31, 145.69]	0.001	17.94 [2.91, 110.76]	0.002	3.93 [0.70, 22.02]	0.119	5.94 [1.40, 25.17]	0.016	4.97 [1.55, 15.93]	0.007	5.77 [1.95, 17.12]	0.002

A category of missing data (MD) was included for variables neutropenia, thrombopenia, *Ascaris*, *Trichuris*, IgG, PCR, but was not significant and is not presented for the sake of clarity

A category of missing data (MD) was included for variables neutropenia, thrombopenia, *Ascaris*, *Trichuris*, IgG, PCR, but was not significant and is not presented for the sake of clarity. *Ref* reference category, *BMI* body mass index, *SCD* sickle cell disease, *RDT* rapid diagnostic test, *HJB* Howell-Jolly Bodies, *PCR* polymerase chain reaction, *US* ultrasound, *CI* confidence interval, *aOR* adjusted odds ratio

^*^Wald global test *P*-value. ^**^ Final model columns only present variables that were retained in multivariable analysis

With *L. loa* MFD as a binary variable, significant correlations persisted between microfilaremia and reduced SV, employing the same modeling approach as in Table 4. Microfilaremic individuals exhibited adjusted *aORs* of 2.12 (95% *CI*: 0.90, 4.99; *P* = 0.084), 1.82 (95% *CI*: 1.05, 3.17; *P* = 0.033), and 1.76 (95% *CI*: 1.22, 2.54; *P* = 0.003) for SV ≤ 80 cm^3^, ≤ 110 cm^3^, and ≤ 150 cm^3^, respectively, compared to amicrofilaremic ones.

Codocytes were observed in one patient with SV ≤ 80 cm^3^, one patient with SV 80–110 cm^3^, and three patients with SV 110–150 cm^3^. Conversely, among the 14 patients with codocytes on blood smear, the median SV was 173 cm^3^ (95% *CI*: 139, 227, missing data: 1).

Regarding the population attributable fraction (PAF) of SV ≤ 80, ≤ 110, and ≤ 150 cm^3^ associated with *L. loa* microfilaremia we found it to be 25.1% (95% *CI*: 1.5, 43.0), 21.2% (95% *CI*: 5.5, 34.3), and 18.8% (95% *CI*: 8.9, 27.6), respectively.

### Spleen parenchymal lesions

We observed 8 individuals (0.8%, 95% *CI*: 0.4, 1.6) presenting with hyperechoic homogeneous macronodules, including 3 cases (0.5%, 95% *CI*: 0.0, 1.4) among amicrofilaremic individuals and 5 cases (1.4%, 95% *CI*: 0.5, 3.3) among microfilaremic individuals (*P* = 0.110). Only 2 hyperechoic micronodules were identified, both of which were found in amicrofilaremic individuals. A total of 8 hypoechoic macronodules (0.8%, 95% *CI*: 0.4, 1.6) were observed across the population, with 2 cases (0.3%, 95% *CI*: 0.03, 1.1) among amicrofilaremic individuals and 6 cases (1.7%, 95% *CI*: 0.6, 3.7) among microfilaremic individuals (*P* = 0.026). We also identified 12 patients with hyperechoic heterogeneous macronodules or ill-defined hyperechoic lesions, representing 1.2% (95% *CI*: 0.7, 2.1) of the population. Of these, 7 cases (1.1%, 95% *CI*: 0.4, 2.3) were observed in amicrofilaremic individuals, and 5 cases (1.4%, 95% *CI*: 0.5, 3.3) in microfilaremic individuals (*P* = 0.427). The patient with probable Gamna-Gandy bodies had ascites, compatible with underlying cirrhosis (Table 5).

**Table 5:** Factors associated with the presence of any spleen lesion in univariate and multivariable analysis

Variable	Saturated model		Final model	
	*aOR* [95% *CI*]	*P*	*aOR* [95% *CI*]	*P*
Age	1.02 [0.99, 1.04]	0.210		
Sex (Ref.: female)	0.37 [0.18, 0.75]	0.006	0.36 [0.19, 0.70]	0.003
BMI	0.97 [0.87, 1.09]	0.634		
History of neurological malaria (Ref.: No)	0.84 [0.28, 2.53]	0.758		
HbAS SCD (Ref.: HbAA)	1.12 [0.51, 2.45]	0.770		
Neutropenia	0.56 [0.16, 1.99]	0.370		
Lymphopenia	-	–		
*Ascaris lumbricoides* (Ref.: absence)	0.91 [0.39, 2.13]	0.834		
*Trichuris trichiura* (Ref.: absence)	1.14 [0.46, 2.84]	0.775		
Number of Eye worm episodes (Ref.: 0)		0.877^*^		
1–5	1.24 [0.49, 3.16]	0.653		
6–10	1.20 [0.43, 3.36]	0.723		
> 10	0.52 [0.10, 2.56]	0.418		
MD	0.92 [0.20, 4.18]	0.918		
Pan-IgG-RDT loiasis (Ref.: 0)		0.276^*^		
1–5	3.57 [0.45, 28.06]	0.227		
> 5	1.97 [0.21, 18.31]	0.550		
HBJ (Ref. absence)	0.59 [0.20, 1.73]	0.334		
Anti-*Plasmodium* IgG titer (Ref.: < 50 µg per ml)		0.085^*^		0.057^*^
50–70	3.00 [1.03, 8.69]	0.043	3.31 [1.20, 9.12]	0.021
70–85	1.49 [0.44, 5.05]	0.526	1.69 [0.53, 5.36]	0.371
> 85	0.99 [0.29, 3.38]	0.983	1.20 [0.36, 3.92]	0.760
*Plasmodium* PCR (Ref.: absence)	1.17 [0.59, 2.30]	0.654		
Spleen volume (per cm^3^)	1.002 [1.0003, 1.004]	0.019	1.002 [1.0001, 1.003]	0.036
*L. loa* Microfilarial density (Ref.: 0 mf per ml)		0.030^*^		0.013^*^
1–2000	0.85 [0.27, 2.66]	0.78	0.73 [0.24, 2.21]	0.588
2001–8000	3.95 [1.55, 10.03]	0.004	3.62 [1.51, 8.67]	0.004
8001–30,000	2.76 [0.92, 8.26]	0.070	2.93 [1.11, 7.70]	0.029
> 30,000	2.80 [0.31, 25.46]	0.361	2.44 [0.30, 19.84]	0.402

A category of missing data (MD) was included for variables neutropenia, thrombopenia, *Ascaris*, *Trichuris*, IgG, PCR, but was not significant and is not presented for the sake of clarity. *Ref* reference category, *BMI* body mass index, *SCD* sickle cell disease, *RDT* rapid diagnostic test, *HJB* Howell-Jolly Bodies, *PCR* polymerase chain reaction, *US* ultrasound, *CI* confidence interval, *aOR* adjusted odds ratio

^*^Wald global test *P*-value

## Discussion

This study provides a comprehensive description of the bi-dimensional sizes and volume of the spleen and the factors influencing its dimensions variations in a rural population in Central Africa. Notably, we demonstrated for the first time the impact of *L. loa* MFD on splenic volume, and provide a global overview of the respective roles of loiasis and malaria on spleen dimensions with estimates suggesting that *L. loa* mf may have a great influence on splenic dimensions.

A strength of this study lies in the consideration of various comorbidities known to be associated with splenic involvement, including parasitic infections such as malaria, sickle cell disease, and hematological comorbidities. Our findings are consistent with the existing literature regarding the physiological influence of BMI and sex on SV and the splenic involution with age. Additionally, we tried to account for numerous common exposure factors in this region, such as STH and other parasites (*O. volvulus*, *M. perstans*, *S. haematobium*), but given the absence of very low number of cases, their impact should still be investigated in further studies. The fact that no case of schistosomiasis was found is probably due to the low prevalence of this disease in the study area, as shown by surveys conducted on school-age children in 2015 (prevalence of 3% in the Sibiti Health District) and to the fact that praziquantel is available for individual treatment in the health structures. Regarding hookworm infections, our results might be due to technical issues, even if the microscopists were experienced.

Regarding the abnormalities observed in our adult population, chronic malaria exposure, as indicated by *Plasmodium* IgG levels, was positively and gradually associated with splenomegaly, and age. Subsequently, we sought to categorize individuals with the HMS entity. HMS are mainly based on an ancient definition by Fakunle et al. [31] although it has been questioned [32]. Apart from splenomegaly and reduction of splenic volume following antimalarial treatment, it includes several biologic abnormalities, for which different threshold can be found across the literature. When used, the cut-off for total plasmatic IgM varies between 2.5 and 3.5 g/L, or + 2 SD above normal value when the latter is known [31, 35]. For anti-*Plasmodium*-IgG levels, one can apply the threshold of > 1/800 when outdated indirect immunofluorescence techniques are applied, otherwise it is most frequently considered relevant when elevated without specification. For this reason, we proposed two sets of criteria—one stringent and one more permissive—to provide a range for this potential issue in this region, and estimated that they represented between 1.4 and 5.3% of our study population. The figures appear close to those found in other African countries [36–41].

Regarding the association with small spleens, particularly those ≤ 80 cm^3^, which have been associated in one study with excess mortality from pneumococcal infections [30], we demonstrated a gradual association with *L. loa* MFD, with high odds ratio (5.8 to 17.9) for individuals with MFD > 30,000 mf/ml, depending on the SV cutoff. Although not all subgroup analyses revealed significant gradient effects, the fact that *aORs* were consistently increased in individuals with microfilaremia, regardless of their MFD, and that the association was highly significant in those belonging to the highest MFD category, supports the hypothesis of a deleterious effect on the spleen. The primary mechanism might be purely mechanical, directly correlated with circulating *L. loa* mf causing chronic splenic microembolization (akin to sickle cell disease) and leading to secondary atrophy of the reticuloendothelial system (RES), and this could be influenced or not by hemostatic disorders consecutive to hypereosinophilia. The impairment of the RES, by circulating immune complexes present in loiasis and a chronic generation of opsonins and other mediators of phagocytosis may also contribute to an RES atrophy.

Nonetheless, it is crucial to interpret our results within the context of an absence of direct translation between AH and functional hyposplenia. Furthermore, while some studies suggest an association between very small spleens and functional impairment, there is also evidence to the contrary, such as the preserved functionality of small spleen implants post-splenectomy, which underscores the complexity of this relationship and the need for cautious interpretation [42–44]. While the presence of HJB strongly indicates splenic impairment, the absence of HJB is not indicative of functional splenic tissue [45]; nevertheless it remains the reference method widely used to assess splenic function. In our study, the clear association between HJB and AH (and its possible gradient effect) reinforces the validity of our results and suggests that those individuals are at higher risk of functional hyposplenia. Given this context, functionality assessment using cytometric measurement of circulating memory B lymphocytes is required in future studies.

Two other results are worth highlighting. First, no association was found between SV and STH or history of eyeworm, which we considered as a proxy for *L. loa* adult worm burden during life. This supports the hypothesis that the impact of *L. loa* on the spleen is due to the presence of blood mf and to the MFD, and not to the number of adult worms present. Second, AH was associated with low loiasis pan-IgG-RDT reactivity. This result should be interpreted cautiously given that the test we have used has been recently investigated by Veletzky et al., and was found to be often falsely negative in individuals with very high *L. loa* MFDs [46]. Therefore, the association between AH and low RDT reactivity might be explained by high MFDs.

We emphasize that our findings highlight an association rather than establish causality. The possibility of reverse causation was considered, with the main hypothesis that functional hyposplenism might arise from a cause unrelated to *L. loa*, and that this condition can induce the presence or an increase in MFD, as seen in primate models of *L. loa* where high MFDs are allowed by splenectomy. However, parasitological surveys conducted over decades show consistent stability in microfilarial distribution in populations, suggesting that such a condition would have a stable prevalence over time and an evenly distribution in central Africa. At the individual level, the MFD is also plateauing after 15 years in individuals and remains stable after. The underlying condition would therefore occur in children or young adults, and would not worsen after adulthood. We cannot identify a condition meeting those criteria (high prevalence stable over decades, absence of worsening after adulthood) to the best of our knowledge. Nevertheless, we plan future studies with measurements of memory B lymphocytes, monitoring of spleen size over time, including HIV status, and assessing bacterial infection trends in relation with spleen dimension evolution over time to further elucidate these possibilities. While splenectomy is associated with increased MFD due to complete loss of splenic filtration, functional hyposplenia represents a partial reduction in splenic activity rather than its complete absence. This may explain why an increase in mf/ml is not observed, as compensatory or adaptative mechanisms could mitigate the effects of reduced splenic function.

Lastly, we estimated that nearly a quarter of the small spleen burden in our study population could be attributable to detectable microfilaremia. Although we mostly found hyperechoic macronodular rather than micronodular lesions—in relation with the use of a convex probe as detailed in the limits thereafter—, we observed an association between MFD and the presence of US anomalies, providing indirect evidence of a potentially chronic deleterious effect of loiasis on the spleen. The only comparable cross-sectional study conducted in an endemic area was performed in Gabon [18]. This study did not find an effect of *L. loa* MFD on spleen dimensions, likely due to the limited sample size (216 participants) and, more critically, the small number of individuals with over 8000 mf/ml (10 participants). In contrast, our study included 97 individuals with more than 8000 mf/ml, and the observed significant differences were predominantly found in this subgroup with intermediate and high MFD levels. Further studies are needed, but we believe the lack of association in the Gabon study is primarily due to insufficient sample size. Regarding lesion characterization, the Gabon study reported hypoechoic macronodules in 3 participants (1.4%, 95% *CI*: 0.3, 4.0), all of whom were microfilaremic (3.6%, 95% *CI*: 0.7, 10.1). Hypoechoic micronodules, visible only with the linear probe, were observed in 3 participants (1.4%, 95% *CI*: 0.3, 4.0), all amicrofilaremic. This corresponds to a prevalence of 2.3% for micronodules among amicrofilaremic individuals. In comparison with our findings, it is noteworthy that the prevalence levels across different lesion types appear relatively consistent, as the confidence intervals overlap or are very similar. Importantly, as in the Gabon study, our only significant difference pertains to participants with hypoechoic macronodules, observed exclusively in microfilaremic individuals. In our study, the prevalence of hypoechoic macronodules was significantly higher among microfilaremic individuals compared to amicrofilaremic ones (1.7% vs. 0.3%, *P* = 0.026). However, given the small number of lesions observed, interpretation of this finding remains limited. As suggested in the Gabon study, hypoechoic lesions may be transient. A limitation of our study is that millimetric lesions or areas potentially corresponding to reticuloendothelial system (RES) atrophy could not be detected with our ultrasound probe. Since our main hypothesis revolves around mechanisms affecting the RES, incorporating CT scans would provide valuable insights. Unfortunately, implementing such imaging techniques in these regions is highly challenging. Future studies should focus specifically on hypoechoic lesions to determine whether they represent true spleen pathology or are merely indirect indicators of microfilarial presence.

Our study has some limitations. Although TBS is a reference technique and was prepared and read by highly experienced technicians in our study, we acknowledge this method has a lower sensitivity compared to concentration techniques, which could result in the underestimation of microfilaremia prevalence of *M. perstans* and *L. loa*. However, despite potential misclassification, our study was able to demonstrate a robust association between MFD and AH, underscoring the validity of our findings. We used a convex probe for ultrasonographic examination. Those low-frequency transducers are generally used for abdominal examination as they offer a better coverage of deep structures. However, we acknowledge that the use of a linear high-frequency probe could have allowed the identification of some additional micronodular lesions given its higher resolution in superficial structures, and that we may have missed some millimetric lesions. Therefore, the use of a linear probe could have enhanced the detection or identification of features indicative of RES atrophy, but the use of both probes was not feasible due to limitations in the availability of human resources time. Although very high microfilaremia (> 30,000 mf/ml) were significantly associated with AH (< 80, 110, 150 cm^3^) in logistic analysis, the association could not be evidenced with the volume as a continuous variable using the linear regression model, likely due to a lack of power.

## Conclusions

This study provides a comprehensive description of factors associated with splenic variation in dimension in a general population living in rural Central Africa. The first one was chronic exposure to malaria, leading to increased spleen volume and in some cases to HMS. The second one was *L. loa* microfilaremia, which was strongly associated with AH, accounting for up to 25% of them. If an association with functional hyposplenia remains to be proven, our results point towards this direction, suggesting that, given the size of this attributable fraction, loiasis may be responsible for a significant proportion of excess mortality following malarial and bacterial infections in Central Africa, particularly for encapsulated germs.

Despite these promising results, further studies are necessary to refine our findings and confirm an association with spleen functionality impairment, particularly through cohort studies, that should incorporate B memory cell typing and evaluate long term outcomes, in particular the risk of developing bacterial and malarial infections.

## Data Availability

Anonymized data will be hosted on the https://dataverse.ird.fr/ server, and its terms of use will be those in force on the hosting site.
